# Rare Case of Large Bowel Injury due to Direct Blunt Trauma to a Preexisting Femoral Hernia

**DOI:** 10.1155/2017/5308027

**Published:** 2017-10-25

**Authors:** C. Tinner, M. Odermatt, P. Villiger

**Affiliations:** Department of Abdominal Surgery, General Hospital Chur, 7000 Chur, Switzerland

## Abstract

We report a case of an 85-year-old man with a known asymptomatic left femoral hernia who was admitted to the emergency ward a few hours after falling from a bicycle and suffering from blunt trauma of the handlebar to the left inguinal region. The clinical findings and a computed tomography (CT) scan detecting free air in the femoral hernia sac suggested bowel perforation. Emergency laparotomy 6 hours after the incident confirmed a tear of the sigmoid colon accompanied by free blood and faeces in the left inguinal region of the abdomen. A segmental sigmoid resection and a primary end-to-end colorectal anastomosis were performed. The postoperative course was complicated by delayed oral feeding, a local infection, and a partial left testicle necrosis that led to secondary resection. The patient was discharged after 32 days of in-hospital care. Three months post trauma, we recorded a restitutio ad integrum. The case exemplifies that blunt trauma to preexisting femoral hernias may cause potentially lethal bowel perforation and that the time interval between time of injury and surgical treatment may be a prognostic factor. CT scans seem most suitable for ruling out bowel perforation. The scarce literature for blunt trauma to hernias is reviewed.

## 1. Introduction

Groin hernias are common, with an estimated prevalence of between 5 and 10 percent [1]. Although femoral hernias account for less than 10 percent of groin hernias, they occur more often than inguinal hernias associated with complications like incarceration and strangulation [2]. Femoral hernias are frequently found in elderly women, whereas inguinal hernias are more common in men [3]. On clinical examination, a groin hernia is easily detected, but diagnosis of a femoral hernia is more difficult and is more often confirmed during surgery. Symptomatic hernias are commonly treated surgically. However, considering that severe complications such as incarceration are uncommon, not all patients with asymptomatic hernias profit equally from surgical treatment. For patients with a high operative risk, conservative treatment may be a viable alternative. Guidelines for optimal surgical treatment have been published by the European Hernia Society [4, 5]. As femoral hernias are more prone to complications like incarceration, there is a general consensus that surgical repair should be the standard of care at an early stage [2]. Many patients with asymptomatic femoral hernias are unaware of their potential risks and do not seek medical advice. Therefore, femoral hernias are eventually diagnosed when becoming symptomatic and often require emergent admission and treatment. Very unusual, though potentially fatal, is another complication of untreated hernias, namely, the traumatic rupture of bowel trapped in a hernia sac. Only a few cases of traumatic bowel injuries associated with inguinal hernias are described in the literature. This is to our knowledge the first case report of a large bowel perforation due to a trauma to femoral hernia.

## 2. Case Presentation

**Figure 1:**
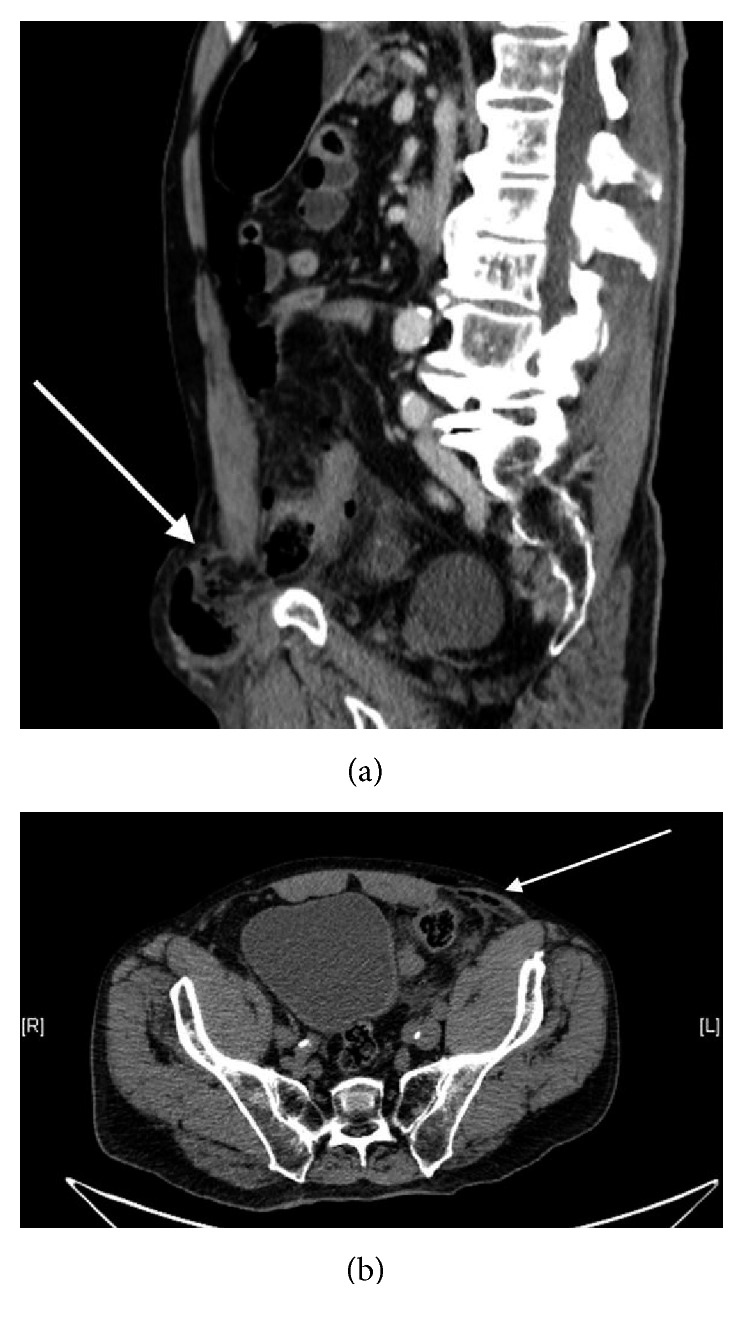
Femoral hernia with free air in preoperative CT: (a) sagittal and (b) axial.

An 85-year-old patient with a medical history of appendectomy, prostatic hyperplasia, and a known but untreated asymptomatic groin hernia had a bicycle accident with a blunt handlebar injury to his left inguinal area. Later on, he complained of pain in the left groin, whereupon his family brought him to the emergency department. He was alert and fully oriented. The abdominal examination revealed diffuse pain, a tense abdomen, and absent bowel sounds. In the left inguinal area, a tennis ball–sized hernia with purplish blue skin discoloration was still visible. Palpation tenderness of the hernia and the lower abdomen was present. The rest of the physical examination was inconspicuous. Laboratory tests revealed no abnormalities (C-reactive protein (CRP) < 1 mg/l, white blood cell count 9.4 × 10^9^/l). A computed tomography (CT) scan detected free air in the femoral hernia sac and abdomen, suggesting bowel perforation (Figure 1()).

The clinical presentation and the CT findings were indicative for laparotomy. Six hours post trauma, emergency laparotomy revealed a circumferential tear of the sigmoid colon accompanied with intra-abdominal perforation. The left inguinal fossa was contaminated with blood and faeces. The subphrenic site and the space between the bowel loops were free of neither faeces nor purulent fluid. A segmental sigmoid resection of 22 cm and a primary end-to-end colorectal anastomosis were performed. Optimal conditions with vital and uncontaminated bowel segments with excellent arterial perfusion justified omitting a diverting stoma. Following extensive lavage, the abdomen was closed leaving a drain. The postoperative course was complicated by delayed oral feeding and a local infection. Ten days after emergency laparotomy, the local conditions needed a surgical revision. The findings included a well-healed anastomosis as well as a partially necrotic left testicle, which had to be removed in an additional inguinal approach. The skin wound healed by secondary intention. With subsequent parenteral nutrition and simultaneous oral feeding, the patient was discharged 32 days after the accident and recovered fully until follow-up three months later.

## 3. Discussion

This case demonstrates that a simple hernia can be complicated by an uncommon and potentially dangerous trauma. In the English literature, we found only few reports on bowel perforation due to blunt trauma in the presence of inguinal hernias (Table 1). High pressure in the hernia sac combined with shearing injury of an irreducible hernia by direct blunt trauma may be causative. Reynolds described in 1995 how incoming and outgoing bowel loops are compressed on direct trauma to an inguinal hernia [14]. In an experimental setup, a pressure of 150–260 mmHg was sufficient to rupture a closed intestinal loop segment. A sudden blunt impact by, for example, an elbow to a gas-filled pouch could generate pressure peaks greater than 300 mmHg [14]. In the present case, the sudden external pressure applied to the sealed loop in the hernia by falling onto the handlebar must have generated sufficient intraluminal pressure to cause perforation. Femoral hernias may be even more prone to such complications than inguinal hernias, as femoral hernias tend to be immobilized in the hernia sac, and they may also less easily slide back than inguinal hernias.

The outcome after surgery for colonic perforation is associated with high morbidity and mortality [15]. The most important determinant for the outcome is time to surgery [16]. Faria et al. analysed the American Society of Anaesthesiologists (ASA) classification, New Injury Severity Score (NISS), and the presence of colonic lesions and identified them as independent prognostic factors for postoperative morbidity. In case of severe faecal peritonitis, colostomy may be considered [17]. Robles-Castillo et al. compared primary closure to colostomy for treatment of traumatic colon injury and concluded that it should be case specific [18]. Chol and Lim showed that laparoscopy is a safe, feasible, and effective procedure for the evaluation and treatment of haemodynamically stable patients with abdominal trauma [19]. However, in cardiopulmonary unstable patients with high probability for intra-abdominal injuries, primary laparotomy is mandatory. Once decided, surgical intervention should occur without delay, as it is crucial to shorten the time for intraperitoneal contamination and associated morbidity.

## 4. Conclusion

Preexisting inguinal or femoral hernias render the bowel more vulnerable to direct physical trauma potentially resulting in a life-threatening bowel perforation. This is one reason more to consider surgical repair in fit patients. In case of groin trauma, even if unspectacular, and subsequent signs of local peritonitis, the possibility of bowel perforation due to a preexisting hernia should be thought of and adequate diagnosis by CT scan should follow.

## Figures and Tables

**Table 1:** Literature overview of bowel perforations due to blunt trauma.

Year, journal	Authors	Title	Treatment; outcome
2014, *Case Reports in Emergency Medicine* [6]	Moustafa et al.	Diagnosis of an inguinal hernia after a blunt inguinal trauma with an intestinal perforation	Laparotomy, resection; discharge on day 10

2012, *Hernia* [7]	Shahin et al.	Small bowel perforation due to blunt trauma to an inguinal hernia: a case report and literature review	Minilaparotomy, suturing of perforation, uneventful; discharge after a few days

2011, *Hernia* [8]	Westwood and Milsom	Colonic perforation following blunt trauma to an inguinal hernia	Laparoscopy, primary closure, uneventful; discharge on day 5

2009, *Hernia* [9]	Neuhaus et al.	Intestinal perforation following a blunt abdominal trauma in patients with pre-existing inguinal hernia	3 cases, laparotomy, 2x uneventful, 1x ileus, and incisional hernia

2009, *Hernia* [10]	Ersoz et al.	Isolated terminal ileum perforation after a kick blow to an inguinal hernia	Direct suturing of perforation, postoperatively uneventful; discharge on day 7

2003, *Hernia* [11]	Oncel et al.	Small bowel perforation due to blunt trauma directly to the inguinal region: a case report	Laparotomy, resection and anastomosis, uneventful; discharge on day 5

2000, *American Journal of Roentgenology* [12]	Uppot et al.	Intestinal perforation from blunt trauma to an inguinal hernia	Laparotomy, colon resection

2000, *Praxis* [13]	Nussbaumer et al.	Traumatic perforation of the small intestine—a rare complication of inguinal hernia	Laparoscopy, direct suturing of perforation
